# Long-acting growth hormone for treating growth hormone deficiency in children: a meta-analysis of randomized controlled trials focusing on changes in body mass index

**DOI:** 10.1210/clinem/dgag093

**Published:** 2026-03-05

**Authors:** Lucie Levaillant, Natacha Bouhours-Nouet, Fabienne Emeriau, Aurelie Donzeau, Stéphanie Rouleau, Carine Villanueva, Kevin Perge, Cécile Teinturier, Sarah Castets, Agnès Linglart, Rachel Reynaud, Regis Coutant

**Affiliations:** Department of Pediatric Endocrinology, University Hospital of Angers, 49000 Angers, France; Reference Center for Rare Pituitary Diseases HYPO, University Hospital of Angers, 49000 Angers, France; Department of Pediatric Endocrinology, University Hospital of Angers, 49000 Angers, France; Reference Center for Rare Pituitary Diseases HYPO, University Hospital of Angers, 49000 Angers, France; Department of Pediatric Endocrinology, University Hospital of Angers, 49000 Angers, France; Reference Center for Rare Pituitary Diseases HYPO, University Hospital of Angers, 49000 Angers, France; Department of Pediatric Endocrinology, University Hospital of Angers, 49000 Angers, France; Department of Pediatric Endocrinology, University Hospital of Angers, 49000 Angers, France; Department of Pediatric Endocrinology, Hospices civils de Lyon, 69500 Bron, France; Reference Center for Rare Pituitary Diseases HYPO, Hospices civils de Lyon, 69500 Bron, France; Department of Pediatric Endocrinology, Hospices civils de Lyon, 69500 Bron, France; Reference Center for Rare Pituitary Diseases HYPO, Hospices civils de Lyon, 69500 Bron, France; Department of Pediatric Endocrinology, Bicetre University Hospital, 94270 Le Kremlin-Bicêtre, France; Reference Center for Rare Pituitary Diseases HYPO, Bicetre University Hospital, 94270 Le Kremlin Bicêtre, Paris, France; Department of Pediatric Endocrinology, La Timone Children’s Hospital, APHM, 13385 Marseille, France; Reference Center for Rare Pituitary Diseases HYPO, La Timone Children’s Hospital, APHM, 13385 Marseille, France; Department of Pediatric Endocrinology, Bicetre University Hospital, 94270 Le Kremlin-Bicêtre, France; Reference Center for Rare Pituitary Diseases HYPO, Bicetre University Hospital, 94270 Le Kremlin Bicêtre, Paris, France; Department of Pediatric Endocrinology, La Timone Children’s Hospital, APHM, 13385 Marseille, France; Reference Center for Rare Pituitary Diseases HYPO, La Timone Children’s Hospital, APHM, 13385 Marseille, France; Department of Pediatric Endocrinology, University Hospital of Angers, 49000 Angers, France; Reference Center for Rare Pituitary Diseases HYPO, University Hospital of Angers, 49000 Angers, France

**Keywords:** growth hormone deficiency, height, body mass index, long-acting growth hormones, children

## Abstract

**Backgound:**

The purpose of this study is to compare the effect of long-acting growth hormone (LAGH) to daily GH on body mass index (BMI) in children with growth hormone deficiency (GHD).

**Methods:**

We searched the PubMed database from its inception to July 2025 and identified three relevant randomized controlled trials lasting over 6 months, with extension phases providing longitudinal BMI data. Longitudinal BMI data were available for lonapegsomatropin, somatrogon, and somapacitan, but not for polyethylene glycol LAGH.

**Results:**

A total of 585 patients were included in the present analysis, of which 346 were in the LAGH group, and 239 were in the daily rhGH group, derived from seven original articles and two abstracts/ePosters. At 12 months, there was a significant difference in BMI SD scores between LAGH and daily GH groups (Mean difference [MD] 0.66 SDS [95% confidence interval [CI] 0.04–1.29]). BMI SDS significantly increased in the LAGH group (MD + 0.41 SDS [95% CI 0.04–0.77] from 0 to 12 months), whereas it did not change in the daily GH group (MD −0.35 SDS [95% CI −0.76 to +0.07] from 0 to 12 months). Between 12 and 24 months, after switching from daily GH to LAGH (daily GH/LAGH), or pursuing LAGH (LAGH/LAGH) in the extension phases of the studies, BMI SDS significantly increased in the daily GH/LAGH switching group (MD + 0.75 SDS [95% CI 0.24–1.27] from 12 to 24 months), whereas it remained steady in the LAGH/LAGH group. Omitting one study at a time from the meta-analysis did not materially affect the results.

**Conclusion:**

An increase in body mass index SD score is associated with the first year of LAGH use.

The primary treatment for children with growth hormone deficiency (GHD) is daily subcutaneous injections of recombinant human growth hormone (rhGH). To reduce the frequency of injections and improve adherence, long-acting GH (LAGH) formulations with once-weekly dosing have been developed (1-10). Currently, four LAGH formulations are in use or pending approval for children with GHD in various countries, following randomized controlled trials (RCTs) (6, 10-19). These include somapacitan (Sogroya®), lonapegsomatropin (Skytrofa®), somatrogon (NGENLA®), and a polyethylene glycol LAGH (Jintrolong®). Published RCTs comparing these LAGH to daily rhGH have shown similar effectiveness in promoting growth and a comparable safety profile to daily rhGH (6, 10-19). Some results regarding body mass index (BMI) changes in these RCTs have also been shared, generally indicating a surprising increase in BMI with LAGH compared to daily rhGH, but these findings either did not reach significance or were not emphasized in the published studies (13, 15-17). So far, there has been no meta-analysis of LAGH in children with GHD specifically focusing on body mass index. Therefore, the present meta-analysis aims to investigate the change in body mass index with the use of these various LAGH therapies compared to daily rhGH for treating children with GHD.

## Methods

This meta-analysis adhered to the “Preferred Reporting Items for Systematic Reviews and Meta-Analyses (PRISMA)” guidelines (20) and was registered in the International Prospective Register of Systematic Reviews (PROSPERO registration number: CRD420251128908).

### Data sources and search strategy

A search was conducted for articles published from inception to July 2025 in PubMed (via the EndNote interface) to identify relevant English articles using the following search strings: “growth hormone deficiency,” “jintrolong,” “somapacitan,” “Somatrogon,” “lonapegsomatropin,” “rhGH-PEG,” “long-acting growth hormone,” “daily growth hormone,” “short-acting growth hormone”, “randomized”, and “child”. Published reports, including abstracts and ePosters from international annual meetings such as those of the Pediatric Endocrine Society (PES), the European Society for Pediatric Endocrinology (ESPE), and those of the Endocrine Society (ENDO), were also hand searched on the web for complementary information regarding the published RCTs.

### Study selection

Studies were included if they met the following criteria: (1) a randomized controlled trial (RCT) lasting more than 6 months; (2) involving children with growth hormone deficiency; (3) receiving LAGH; (4) compared to daily rhGH; and (5) available longitudinal BMI data. Studies reporting the extension phase of these RCTs were also included if they provided BMI data.

### Data extraction and quality assessment

The following data were extracted from the included studies and compiled into a spreadsheet: sample size, patient age and sex, treatment characteristics (intervention and control arms), and outcomes. To reduce potential assessment bias, two independent reviewers selected studies and conducted data extraction. Any disagreements encountered during extraction were resolved through discussion. The methodological quality of the included randomized controlled trials was assessed using the Jadad score (Oxford quality scoring system), which evaluates randomization, blinding, and description of withdrawals/dropouts (score range 0-5; higher scores indicate better quality). Trials scoring ≥3 were considered high quality (21). Publication bias was not formally assessed using Begg's or Egger's tests because the meta-analysis included fewer than 10 studies, and such tests are underpowered and unreliable when based on a small number of trials.

### Data synthesis and statistical analysis

The primary endpoint of the study was body mass index standard deviation score (BMISDS) (CDC reference) (22) and height standard deviation score (HSDS). Given that BMI and height were expressed as a standard deviation score (SDS) in all studies, effect sizes were synthesized as mean differences (MDs), which are directly comparable and clinically interpretable, rather than as standardized mean differences.

Heterogeneity was assessed using the inconsistency index (I^2^), which describes the percentage of total variation across studies attributable to heterogeneity rather than chance, the Cochran's Q test, and the between-study variance (τ^2^). It was also visually inspected through Galbraith plots.

A random-effects model was fitted using the restricted maximum likelihood (REML) method, with the truncated Hartung–Knapp–Sidik–Jonkman (HKSJ) adjustment for confidence intervals, given the small number of included studies.

BMI and Height SD scores at 12 months were compared between LAGH and daily rhGH subjects. CDC growth charts for non-obese children were used (22). BMI and Height SD scores at 24 months (extension phase) were compared between subjects who pursued LAGH (LAGH/LAGH) and those who switched from daily rhGH to LAGH (Switch).

Height and BMI SD scores were compared between baseline (M0) and 12 months (M12) in the LAGH and daily GH groups, respectively, as well as between 12 months (M12) and 24 months (M24) in the LAGH/LAGH groups and daily GH/LAGH groups (switch from daily to LAGH at 12 months), respectively. Since the correlation coefficients between values at M0 and M12, and M12 and M24, together with the standard deviations of the paired differences, were not available, the analysis was conducted under the assumption that the values were uncorrelated. This conservative approach yields wider confidence intervals than the true ones, thereby reducing statistical power but without increasing the risk of a false-positive difference.

Sensitivity analyses were performed using a leave-one-out approach at the study level, whereby each study was sequentially removed from the model along with all its associated effect sizes. This analysis was intended to evaluate whether the direction of the overall effect was unduly driven by any single study.

All statistical analyses were performed using Stata/MP version 18 (StataCorp LLC, College Station, TX, USA).

## Results

### Selection of eligible articles

The initial electronic search yielded 910 results. Articles were further screened based on the title and abstract to determine eligibility. Upon full-text screening of these articles, RCTs were considered eligible for final analysis. Fig. S1 (23) represents the study flow diagram.

### Study characteristics

Three English articles describing the RCTs (10, 11, 13) and four English articles about the extension phase (switching from daily rhGH to LAGH in the daily rhGH group, and pursuing LAGH in the LAGH group) (15-17, 24) were included in the final analysis, along with two abstracts and ePoster about the extension phase of the third RCT (25, 26) (Table 1). None of the phase II dose-finding studies were included because longitudinal BMI data were unavailable for three LAGH trials (4-6, 8) (Table 1). Although the phase II study evaluating lonapegsomatropin reported some longitudinal BMI-related outcomes, the absence of SD scores for BMI data precluded its inclusion in the meta-analysis (3) (Table 1). All data sources were mutually exclusive, ensuring that no patient was included more than once in the same analysis. The Jadad score for the included RCTs was 3, indicating good quality. A total of 585 patients were included in the present analysis, of which 346 were in the LAGH group and 239 were in the daily rhGH group.

**Table 1:** Characteristics of the selected studies

Authors and year	Study design	Intervention (I) Drug/Dose	Comparator (C) Drug/Dose	Patients randomized (N)	Age at treatment initiation (year)	Treatment duration
Phase 3 trials						
Thornton et al 2021 (8)	Randomized, multicenter, open-label, controlled, parallel group phase 3 trial	Lonapegsomatropin 0.24 mg/kg/wk	Daily GH 0.24 mg/kg/wk	I: 105 C: 56	I: 8.5 ± 2.7 C: 8.5 ± 2.8	52 weeks
Miller et al 2022 (11)	Randomised, multicenter, open-label, controlled, parallel group phase 3 trial	Somapacitan 0.16 mg/kg/wk	Daily GH 0.24 mg/kg/wk	I: 132 C: 68	I: 6.4 ± 2.2 C: 6.4 ± 2.4	52 weeks
Deal et al 2022 (9)	Randomized, multicenter, open-label, controlled, parallel-group, phase 3 trial	Somatrogon 0.66 mg/kg/wk	Daily GH 0.24 mg/kg/wk	I: 108 C 114	I: 7.83 (3.01-11.96) C: 7.61 (3.05-11.85)	52 weeks
Maniatis et al 2022 (13) and 2025 (22)	Extension phase of a randomized, multicenter, open-label, controlled, parallel group phase 3 trial	Lonapegsomatropin 0.24 mg/kg/wk	Switch from daily GH to lonapegsomatropin 0.24 mg/kg/wk	I: 100 C: 54		> 52 weeks
Miller et al 2023 (14) and 2025 (15)	Extension phase of a randomized, multicenter, open-label, controlled, parallel group phase 3 trial	Somapacitan 0.16 mg/kg/wk	Switch from daily GH to somapacitan 0.16 mg/kg/wk	I: 127 C: 67		> 52 weeks
Magnie et al 2023 (23) (ePoster)	Randomized, multicenter, open-label, controlled, parallel-group, phase 3 trial	Somatrogon 0.66 mg/kg/wk	Daily GH 0.24 mg/kg/wk	I: 108 C: 114	I: 6.4 ± 2.2 C: 6.4 ± 2.4	52 weeks
Wajnrajch et al 2021 (24) (abstract)	Extension phase of a randomized, multicenter, open-label, controlled, parallel group phase 3 trial	Somatrogon 0.66 mg/kg/wk	Switch from daily GH to somatrogon 0.66 mg/kg/wk	I: 108 C: 114		> 52 weeks
Phase 2 dose-finding studies excluded owing to absent or insufficient BMI evolution during treatment						
Chatelain et al 2017 (3)	Randomised, multicenter, open-label, controlled, parallel group phase 2 trial	Lonapegsomatropin 0.14, 0.21, or 0.30 mg/kg/wk	Daily GH 0.21 mg/kg/wk	I: 40 C: 13		26 weeks
Zelinska et al 2017 (4) and Zadik et al 2023 (5)	Randomised, multicenter, open-label, controlled, parallel group phase 2 trial	Somatrogon 0.25, 0.48, or 0.66 mg/kg/wk	Daily GH 0.24 mg/kg/wk	I: 42 C: 11		52 weeks
Luo et al 2017 (6)	Randomised, multicenter, open-label, controlled, parallel group phase 2 trial	PEGylated recombinant human growth hormone 0.1 or 0.2 mg/kg/wk	Daily GH 0.25 mg/kg/wk	I: 63 C: 34		25 weeks
Savendahl et al 2020 (8)	Randomised, multicenter, open-label, controlled, parallel group phase 2 trial	Somapacitan 0.04, 0.08, or 0.16 mg/kg/wk	Daily GH 0.24 mg/kg/wk	I: 45 C: 14		26 weeks

### Heterogeneity analysis of baseline characteristics

There was no heterogeneity between studies for the baseline parameters age (I^2^ = 0%, τ^2^ = 0.00, Cochran's Q test *P* = .84) and body mass index SDS (I^2^ = 0%, τ^2^ = 0.00, Cochran's Q test *P* = .49). Heterogeneity was observed for height SDS (I^2^ = 72%, τ^2^ = 0.068, Cochran's Q test *P* = .04)(Fig. S2) (23), but the studies were kept in the present analyses, which focused on BMI. The baseline parameters did not differ between the LAGH and daily rhGH groups in the included studies (Fig. S3) (23).

### Effect of LAGH compared to daily rhGH on height SDS and BMI SDS

**Figure 1:**
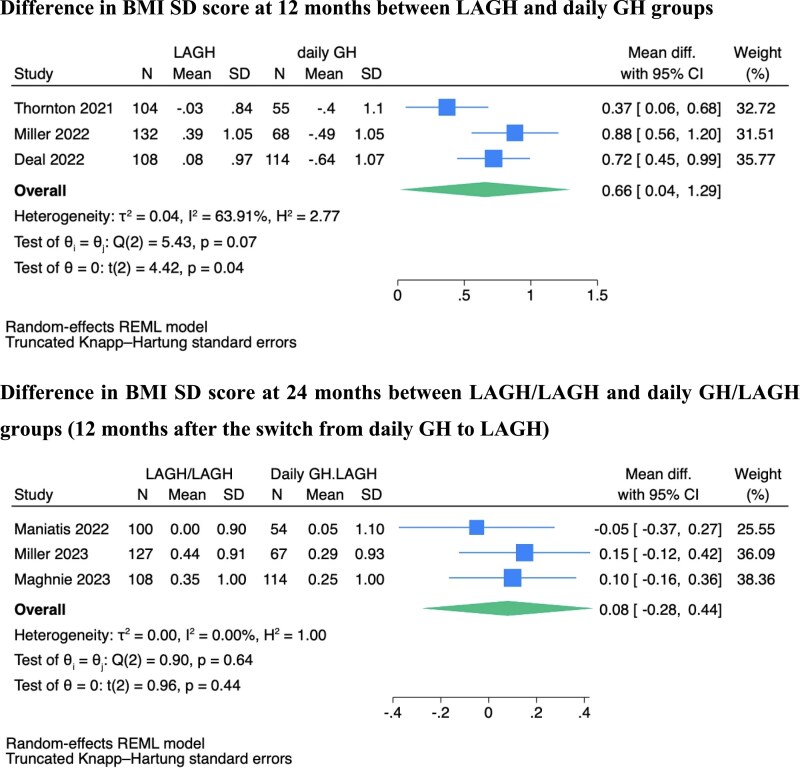
Forest plot comparing BMI SD score between LAGH and daily GH groups at 12 months, and between LAGH/LAGH and daily GH/LAGH groups at 24 months (12 months after the switch from daily GH to LAGH).

**Figure 2:**
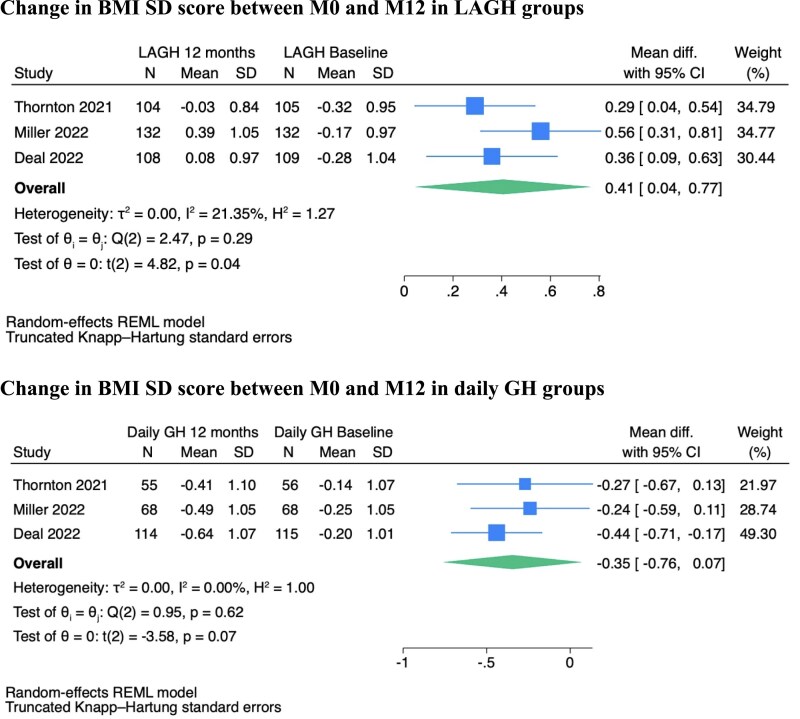
Forest plot showing the changes in BMI SD score between 0 and 12 months in the LAGH and the daily GH groups.

At 12 months, there was a significant difference in BMI SD scores between LAGH and daily rhGH groups (Mean difference [MD] 0.66 SDS [95% confidence interval [CI] 0.04–1.29]), indicating a higher BMI gain in the LAGH group (Fig. 1). BMI SDS significantly increased between 0 and 12 months in the LAGH group (MD 0.41 SDS [95% CI 0.04–0.77]), whereas it did not change in the daily rhGH group (MD −0.35 SDS [95% CI −0.76 to +0.07]) (Fig. 2).

**Figure 3:**
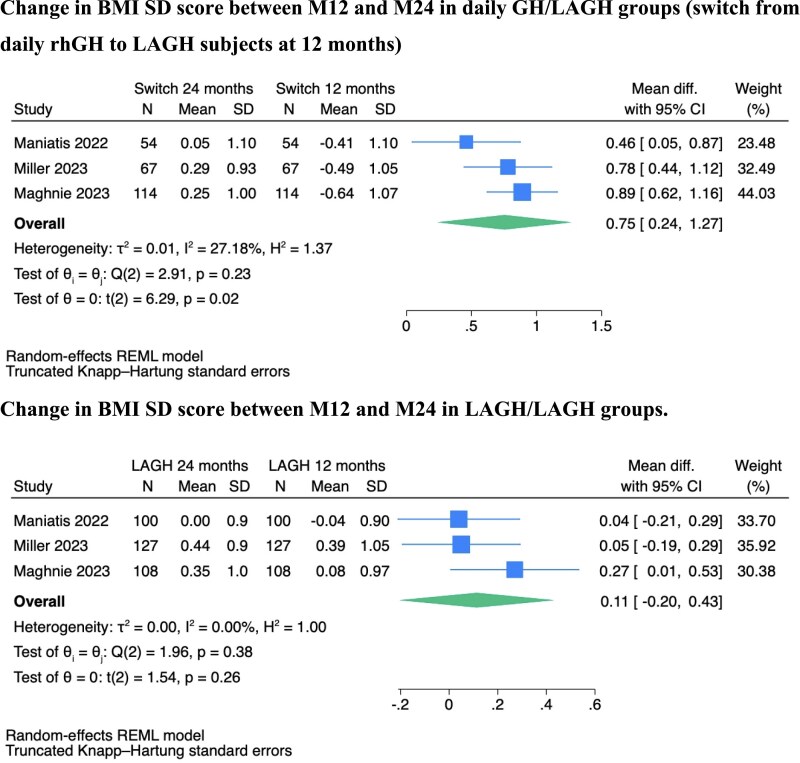
Forest plot showing the changes in BMI SD score between 12 and 24 months in the daily GH/LAGH groups (after the switch from daily GH to LAGH) and in the LAGH/LAGH groups.

At 24 months, which is 12 months after switching from daily GH to LAGH (daily GH/LAGH) or continuing LAGH (LAGH/LAGH) during the extension phases of the studies, both BMI and height SD scores were similar in the two groups (Fig. 1 and Fig. S4) (23). This indicates that the daily GH/LAGH group experienced an increase in BMI SD score between 12 and 24 months after the switch (MD 0.75 SDS [95% CI 0.24–1.27]), while the LAGH/LAGH group maintained steady BMI SD scores during that period (Fig. 3).

Omitting one study at a time from the meta-analysis did not materially affect the results.

Height SD scores and changes in Height SD scores are displayed in Figs. S4-6 (23). No differences were observed between the LAGH and daily GH groups for Height SD scores.

## Discussion

To the best of our knowledge, this is the first study to perform a meta-analysis of BMI changes following LAGH treatment for GHD in children. While height SD scores were similar after 1 year between the LAGH and daily rhGH groups, the LAGH group had an approximately 0.66 SD higher BMI. Additionally, a similar BMI gain was observed in the year after switching from daily rhGH to LAGH during the extension phases of the randomized controlled trials, while BMI SD scores remained steady in the group continuing LAGH treatment. While the growth-promoting effects of LAGH and daily rhGH were similar, this likely indicates a differential effect on body composition (10, 11, 13, 15-17, 24-26).

This effect of LAGH on BMI appears to be independent of the specific LAGH molecule, as it was observed across all three LAGH types analyzed in this study (somapacitan, somatrogon, and lonapegsomatropin). We could not assess the impact of PEG-LAGH (Jintrolong) because longitudinal BMI data were unavailable in the published study (6). Additionally, we could not determine if this effect on BMI varied by gender because the data were unavailable. Although the exact mechanism behind the increase in BMI SD score remains unknown, it seems to be a common effect among different LAGH molecules. Body composition studies are needed to determine whether the BMI increase is primarily due to changes in lean or fat mass.

Comparing the LAGH effect in adult populations with GHD may provide useful context (27-29). In randomized trials, the effect of LAGH on body composition (trunk fat and lean body mass) was compared with placebo after 6 to 9 months (27-29), while the effect of daily rhGH was possibly reported (27, 29). Lonapegsomatropin, somatrogon, and somapacitan showed a significant increase in lean body mass compared to placebo (27-29). Lonapegsomatropin and somapacitan, but not somatrogon, showed a significant reduction in trunk fat mass (27-29), resulting in the withdrawal of the application to extend somatrogon approval to adult GHD in the EU (30). Surprisingly, formal statistical comparisons with daily GH were not published for these outcomes. Nevertheless, descriptive analyses suggested a trend toward greater reductions in trunk fat percentage with daily GH compared with lonapegsomatropin (−3% and −1.6%) (27) and somapacitan (−2.5% and −1.2%) (29), and a similar increase in lean body mass (+1.5 kg). No daily rhGH was used in the adult GHD somatrogon study (28). Interestingly, other body composition endpoints were reported in the somapacitan study and showed a difference in favor of daily GH for gynoid fat mass (*P* = .028), and trends in favor of daily GH for total fat mass (*P* = .063) and truncal fat mass (*P* = .075). Equally interesting, a post hoc analysis of the lonapegsomatropin study was performed (27), which retained only subjects with IGF-1 below +1.75 SD at 9 months, as the authors noted that “GH is lipolytic and IGF-1 is adipogenic”. This analysis showed a similar reduction in trunk fat percentage (−2.6% vs −2.4% between daily rhGH and lonapegsomatropin) (27). Overall, these findings suggest that LAGH may yield fewer improvements in body fat than daily GH. However, these differences in adults with GHD were small and may have been related to IGF-1 levels, while long-term adherence to daily GH treatment remains a major challenge.

Indeed, from a pathophysiological perspective, LAGH formulations, compared with daily rhGH, are characterized by a marked dissociation between circulating GH and IGF-1 levels (7, 9). Following LAGH administration, GH concentrations are detectable mainly during the first 48 hours post-injection and subsequently decline, whereas IGF-1 levels peak around 48 hours, remain close to median levels at approximately 96 hours, and decrease to low levels between days 5 and 7 (7, 9, 31, 32). As a result, IGF-1 exposure under LAGH displays substantial intra-week fluctuations, with an approximate 2 SDS difference between the peak at day 2 and the trough at day 7, while IGF-1 levels during daily rhGH administration remain comparatively stable (7, 9, 31, 32). Given that GH and IGF-1 exert distinct effects on fat and lean mass (33), these differences in hormonal kinetics between LAGH and daily rhGH may translate into differential effects on BMI. GH is the primary mediator of lipolysis, and its activity under LAGH is largely limited to the first 48 hours after injection, which may contribute to a reduced lipolytic effect compared with daily GH administration (33). In contrast, GH-induced IGF-1 stimulates muscle protein synthesis (34, 35). Because IGF-1 levels decline only to low concentrations between days 5 and 7, this may explain the sustained effects on lean mass observed with LAGH, which are comparable to those seen with daily GH. It is also possible that the BMI effect of LAGH differs among LAGHs, based on their mechanisms of protraction and target tissue penetration. In this meta-analysis, we used a leave-one-out approach at the study level, in which each study was sequentially removed from the model along with all its associated effect sizes. We found that omitting one study at a time did not materially affect the results. However, we were unable to compare each LAGH with the others. Further studies will be needed to clarify this point.

For comparison, it is informative to consider the effects of recombinant IGF-1 (rhIGF-1) therapy. A recent publication on rhIGF-1 reported that patients with Laron syndrome (autosomal recessive growth hormone receptor defect) experienced an increase in BMI SDS during treatment, from a mean of −0.24 SDS at baseline to approximately +1.2 SDS after 5 years of therapy (36). A similar trend was observed in patients treated with rhIGF-1 without Laron syndrome, with BMI SDS increasing from −0.8 SDS at baseline to 0.3 SDS (36). These findings are consistent with another study reporting a mean increase of 0.86 BMI SDS following rhIGF-1 treatment (37). Collectively, these data suggest that IGF-1 exposure in the absence of an effective GH signal may be associated with an increase in BMI. Earlier reports in small cohorts of IGF-1-treated subjects have also shown an increase in total adipose tissue (38-40).

In addition, GH is known to stimulate appetite; however, the specific effects of long-acting GH (LAGH) formulations on appetite regulation have not yet been systematically evaluated (41). Furthermore, several studies have emphasized the major influence of the household environment and lifestyle behaviors—such as family-based physical activity and dietary habits—on children's body composition (including lean and fat mass) and physical development. Family-based interventions have been shown to effectively increase physical activity, which is closely associated with more favorable body composition trajectories during growth (42). Notably, physical activity and dietary habits were not recorded in the LAGH comparative studies.

The increase in BMI SD score with LAGH in children seems to stabilize over time, as data beyond 2 years of treatment have not shown further increases in BMI (17, 24, 25). No harmful metabolic effects (on glucose or lipid metabolism) have been reported with LAGH (5, 25) compared to daily rhGH, which indicates limited consequences of the changes in body composition.

One limitation of this meta-analysis is the absence of BMI data in several studies. In particular, dose-finding phase 2 trials could not be included because detailed longitudinal BMI data were unavailable. In the dose-finding phase 2 trial of lonapegsomatropin, the mean difference in the change in BMI SD score from baseline to week 26 between children receiving LAGH and those receiving daily GH was +0.69 (SD not indicated) (3), which is very close to the +0.66 observed in the present meta-analysis (3-6, 8). No additional relevant information was identified in the Food and Drug Administration review files for the three submitted LAGH products (43-45).

In conclusion, this meta-analysis highlighted the increase in body mass index associated with the use of LAGH compared to daily rhGH in children with GHD. Further studies are needed to understand the specific effects on lean and fat mass, as well as the mechanisms behind this increase. Although the effect appears to be limited in both duration (with no further increase after 1 year) and magnitude (averaging a 0.66 BMI SD score increase), future studies will help determine if there is a risk profile in GHD children for more significant effects and whether this change in BMI is also seen in other GH indications, such as children with SGA or Prader-Willi syndrome, since these children are susceptible to adverse metabolic and body composition effects.

## Data Availability

Original data generated and analyzed during this study are included in this published article or in the data repositories listed in References.
